# Alveolar Macrophages Play a Key Role in Cockroach-Induced Allergic Inflammation via TNF-α Pathway

**DOI:** 10.1371/journal.pone.0047971

**Published:** 2012-10-19

**Authors:** Joo Young Kim, Jung Ho Sohn, Je-Min Choi, Jae-Hyun Lee, Chein-Soo Hong, Joo-Shil Lee, Jung-Won Park

**Affiliations:** 1 Ewha Womans University College of Pharmacy, Research Institute of Pharmaceutical Sciences, Seoul, South Korea; 2 Department of Life Science, Hanyang University, Seoul, South Korea; 3 Department of Internal Medicine, Yonsei University College of Medicine, Seoul, South Korea; 4 Brain Korea 21 Project for Medical Science, Yonsei University College of Medicine, Seoul, South Korea; 5 Institute of Allergy, Yonsei University College of Medicine, Seoul, South Korea; 6 Center for Immunology and Pathology, Korea National Institute of Health, Osong, South Korea; Louisiana State University Health Sciences Center, United States of America

## Abstract

The activity of the serine protease in the German cockroach allergen is important to the development of allergic disease. The protease-activated receptor (PAR)-2, which is expressed in numerous cell types in lung tissue, is known to mediate the cellular events caused by inhaled serine protease. Alveolar macrophages express PAR-2 and produce considerable amounts of tumor necrosis factor (TNF)-α. We determined whether the serine protease in German cockroach extract (GCE) enhances TNF-α production by alveolar macrophages through the PAR-2 pathway and whether the TNF-α production affects GCE-induced pulmonary inflammation. Effects of GCE on alveolar macrophages and TNF-α production were evaluated using in vitro MH-S and RAW264.6 cells and in vivo GCE-induced asthma models of BALB/c mice. GCE contained a large amount of serine protease. In the MH-S and RAW264.7 cells, GCE activated PAR-2 and thereby produced TNF-α. In the GCE-induced asthma model, intranasal administration of GCE increased airway hyperresponsiveness (AHR), inflammatory cell infiltration, productions of serum immunoglobulin E, interleukin (IL)-5, IL-13 and TNF-α production in alveolar macrophages. Blockade of serine proteases prevented the development of GCE induced allergic pathologies. TNF-α blockade also prevented the development of such asthma-like lesions. Depletion of alveolar macrophages reduced AHR and intracellular TNF-α level in pulmonary cell populations in the GCE-induced asthma model. These results suggest that serine protease from GCE affects asthma through an alveolar macrophage and TNF-α dependent manner, reflecting the close relation of innate and adaptive immune response in allergic asthma model.

## Introduction

German cockroaches are a well-known causative allergen for allergic asthma [1]. It contains several major allergens and proteases. Classically, allergens induce immune responses that lead to Th2 lymphocyte differentiation, production of IgE, and mast cell activation; however, prolonged administration of allergen may induce regulatory T cells and tolerance to the allergens [2]. Recently, another type of allergens were classified as type II allergens, and these allergens bypass normal tolerogenic mechanisms and directly induce allergic diseases by sensitization of local routes [3], [4]. One example of such type II allergens includes the active proteases derived from cockroach, house dust mite, and fungal extracts.

German cockroach extract (GCE) was reported to contain active serine proteases [5], [6], [7]. Serine protease affects the development of inflammation and allergic immune responses through specific receptor systems, such as the protease-activated receptor (PAR)-2 in a variety of cell types [4]. PAR-2, a member of the G protein-coupled receptor family [8], is activated by various serine proteases such as mast cell tryptase [9], trypsin-like enzymes [10], and certain allergens from house dust mites [11] or cockroaches [8], [12]. Serine proteases stimulate the N-terminal exodomain of the receptor and cleave the receptor at this site [13]. Alteration of PAR-2 results in coupling and activation of G proteins, triggers a cascade of signaling events, and thereby leads to intracellular Ca^2+^ influx [14] and tumor necrosis factor (TNF) production. These events contribute to the development of eosinophilic inflammation and airway hyperresponsiveness (AHR) in asthma [4]. Others, however, have reported that PAR-2 may be protective against bronchoconstriction or AHR [15]. Thus, the role of PAR-2 in asthma remains controversial.

**Figure 1:**
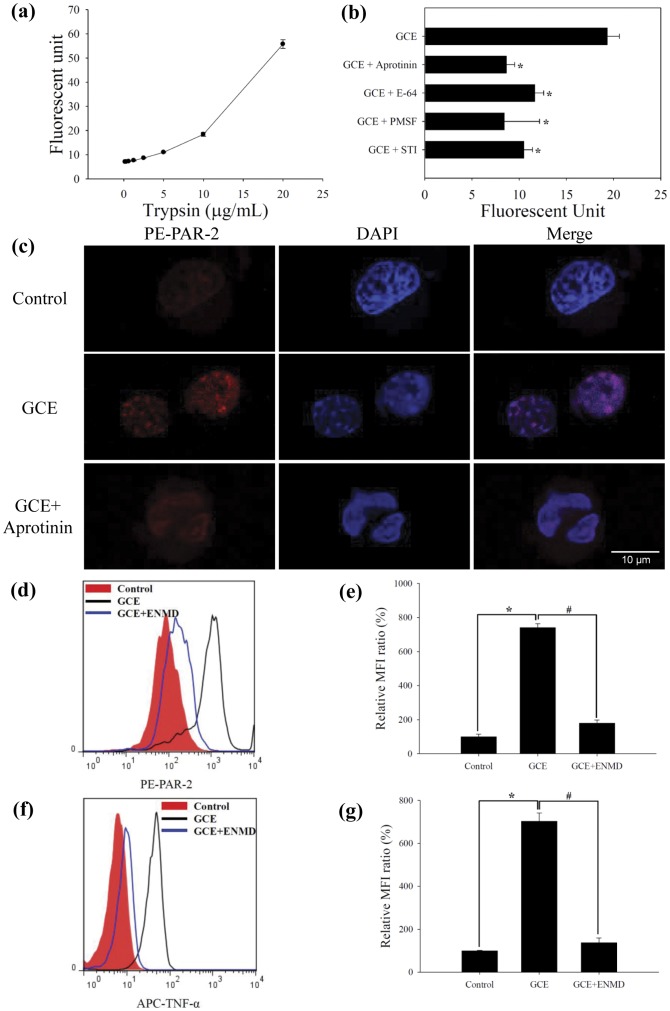
Serine protease of GCE activates PAR-2. (a) Standard curve of trypsin at concentrations ranging from 0.16 to 20 μg/mL. (b) Serine protease activities on FITC-casein. * indicates statistical significance compared with “GCE” (n = 3, *p*<0.05). (c) PAR-2 internalization following activation with GCE and/or aprotinin in MH-S cells. Internalization of the PAR-2 is visualized by confocal imaging of intracellular staining. Intracellular expressions of (d) PAR-2 and (f) TNF-α in MH-S cells incubated with GCE or GCE+ENMD. (e) and (g) are relative MFI ration in “d” and “f” panels, respectively. * indicates statistical significance between “Control” and “GCE” (n = 3, *p*<0.05), ^#^ indicates statistical significance between “GCE” and “GCE+ENMD” (n = 3, *p*<0.05). All data are representative of three independent experiments. *ENMD*, ENMD-1068.

PAR-2-expressing cells, such as alveolar macrophages [13], epithelial cells, mast cells, and fibroblasts are located throughout the airways and encounter inhaled allergens or particles that contain serine protease activity. Alveolar macrophages are able to produce large amounts of TNF-α [4], [16]. Recent studies indicated that depletion of alveolar macrophages [17] or blockade of TNF-α [18] prevents AHR and progressive inflammatory injuries in an ovalbumin-induced asthma model. TNF-α blockade also ameliorates AHR, impairment of lung function, and quality of life in patients with severe asthma [19], [20]. These findings suggest that TNF-α expression by alveolar macrophages may play a key role in allergic inflammation, especially when induced by indoor allergens such as house dust mites, fungus or cockroaches.

**Figure 2:**
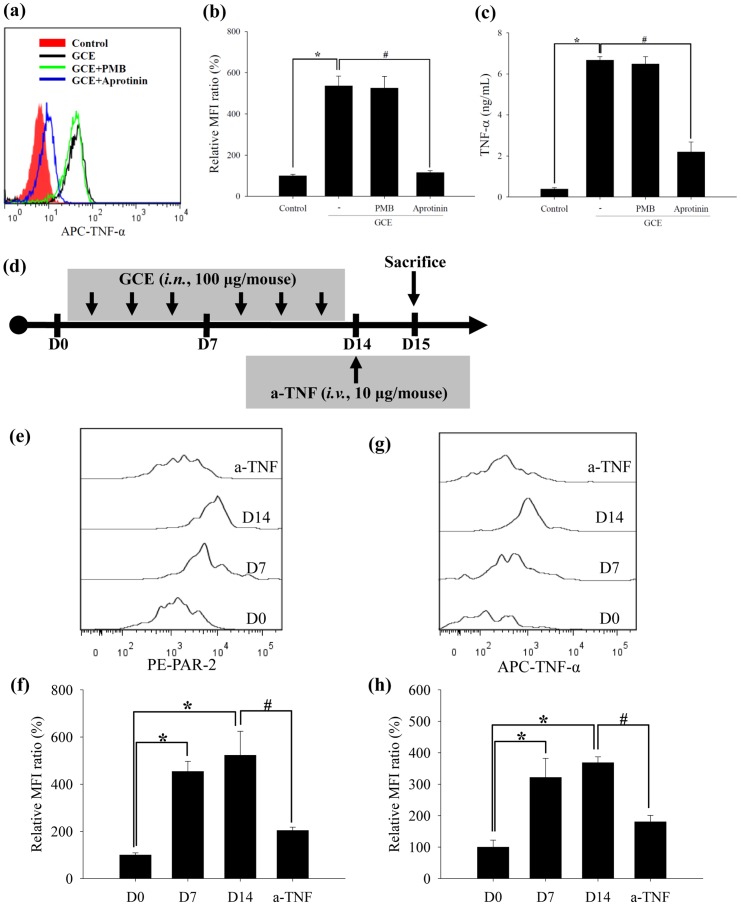
GCE induces TNF-α production in macrophages. (a) TNF-α production in MH-S cells incubated with GCE, GCE+PMB, or GCE+aprotinin. (b) Relative MFI ratio in “a” panel. (c) TNF-α secretion in the culture supernatant of “a” panel. * indicates statistical significance between “Control” and “GCE” (n = 3, *p*<0.05). ^#^ indicates statistical significance between “GCE” and “GCE+Aprotinin” (n = 3, *p*<0.05). (d) Scheme for short-term GCE exposure model. Kinetics of intracellular (e) PAR-2 and (g) TNF-α expression from alveolar macrophages in the BAL fluid of the short-term GCE exposure model. (f) Relative MFI ratio in “e” panel. (h) Relative MFI ratio in “g” panel. * indicates statistical significance to “D0” (n = 5, *p*<0.05). ^#^ indicates statistical significance between “D12” and “a-TNF” (n = 5, *p*<0.05). All data are representative of three independent experiments.

In this study, we investigated whether alveolar macrophages are stimulated by GCE through PAR-2 and whether production of TNF-α by alveolar macrophages plays a key role in the development of GCE-induced allergic inflammation in a mouse asthma model.

**Figure 3:**
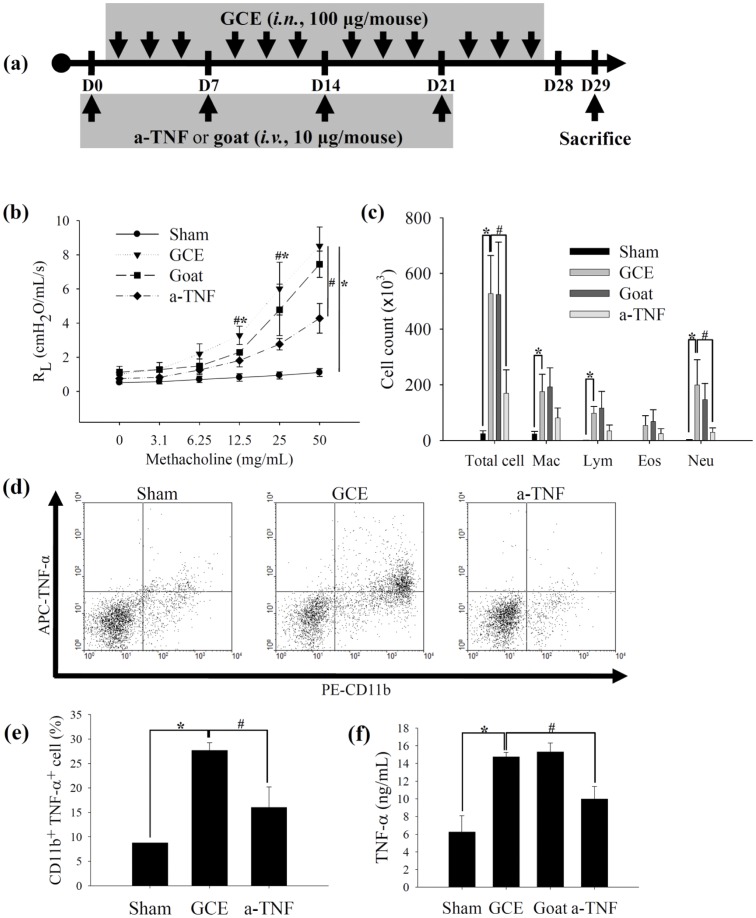
GCE promotes pulmonary inflammation through the TNF-α pathway. (a) Scheme for TNF-α neutralization of GCE-induced asthma model. *a-TNF*, anti-TNF-α mAb; *goat*, goat IgG. (b) AHR and (c) BAL cell count. (d) TNF-α-producing macrophage population in the lung tissue. (e) Quantitative analysis of CD11b^+^TNF-α^+^ cell population from “d” panel. (f) TNF-α levels in lung homogenates. * indicates statistical significance between “Sham” and “GCE” (n = 5, p<0.05). ^#^ indicates statistical significance between “GCE” and “a-TNF” (n = 5, *p*<0.05). All data are representative of three independent experiments. *R_L_*, pulmonary resistance; *Mac*, macrophage; *Lym*, lymphocyte; *Eos*, eosinophil; *Neu*, neutrophil.

## Materials and Methods

### Animals

Female BALB/c Cr Slc mice (6-weeks) were purchased from Japan-SLC (Hamamatsu, Japan). This study was carried out in strict accordance with the recommendations in the Guide for the Care and Use of Laboratory Animals of the Institute of Laboratory Animal Resources Commission on Life Sciences National Research Council, USA. The protocol was approved by Institutional Animal Care and Use Committee (08–183) in Yonsei University College of Medicine (Seoul, Korea), which has been fully accredited by the Association for Assessment and Accreditation of Laboratory Animal Care, International.

Mice exposed intranasally to GCE (100 μg/mouse) 3 times per week for 2 or 4 weeks (short- and long-term GCE exposure models, respectively).

**Figure 4:**
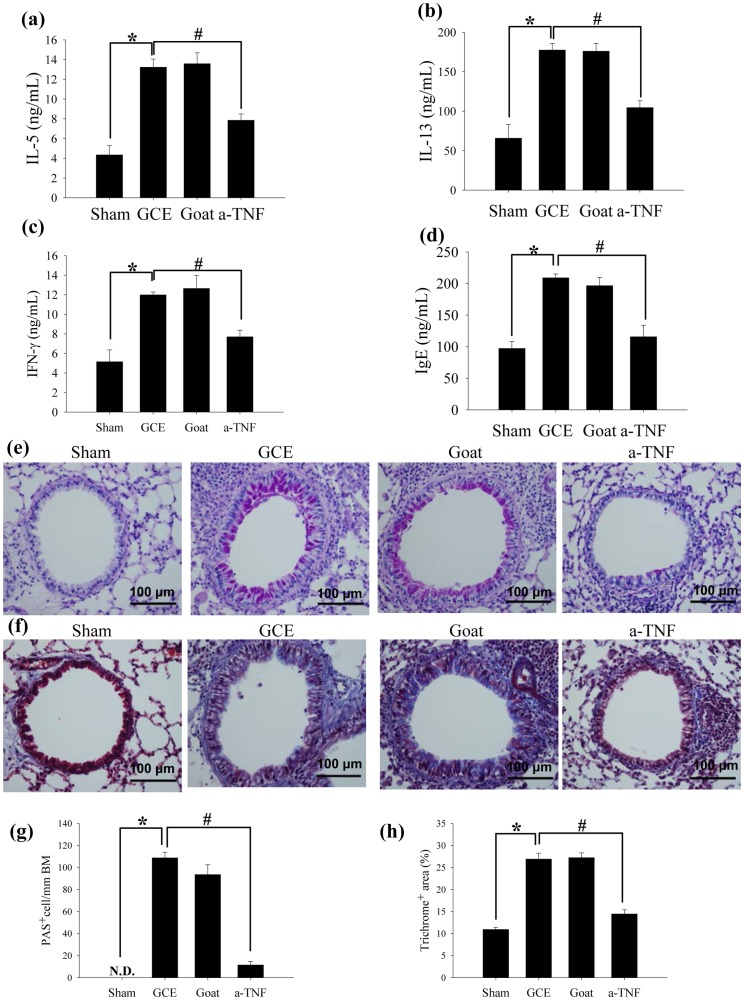
GCE promotes allergic phenotype through the TNF-α pathway. Lung homogenates were harvested and used for measuring (a) IL-5, (b) IL-13 and (c) IFN-γ. (d) The level of serum IgE in the blood. Lung tissues were stained with (e) PAS and (f) Masson's Trichrome. (g) PAS-positive cells in peri-bronchial regions and (h) total collagen deposition in the lung tissue were quantitatively calculated. * indicates statistical significance between “Sham” and “GCE” (n = 5, *p*<0.05). ^#^ indicates statistical significance between “GCE” and “a-TNF” (n = 5, *p*<0.05). All data are representative of three independent experiments.

### GCE preparation

GCE was prepared as previously described [7]. Fifty grams of frozen German cockroaches were homogenized in liquid nitrogen and defatted in 200 mL of ethyl ether and 200 mL of ethyl acetate. Extractions were preformed with slow stirring at 4°C overnight in PBS containing 6 mmol/L 2-mercaptoethanol and 1 mg/mL 1-phenyl-3-(2-thiazolyl)-2-thiourea to prevent melanization. The extract was then centrifuged at 10,000×*g* for 30 minutes at 4°C, and the supernatant was finally filtered through 0.2-μm filters. The endotoxin from the supernatant was removed by the Detoxi-Gel^TM^ Endotoxin Removing Gel (Pierce Biotechnology, Rockford, IL). The endotoxin from the GCE was removed and measured to be below 0.01 EU/mL by the chromogenic Limulus Amebocyte Lysate test (Lonza, Walkersville, MD). The GCE contained 0.11 U/mg Bla g1.

**Figure 5:**
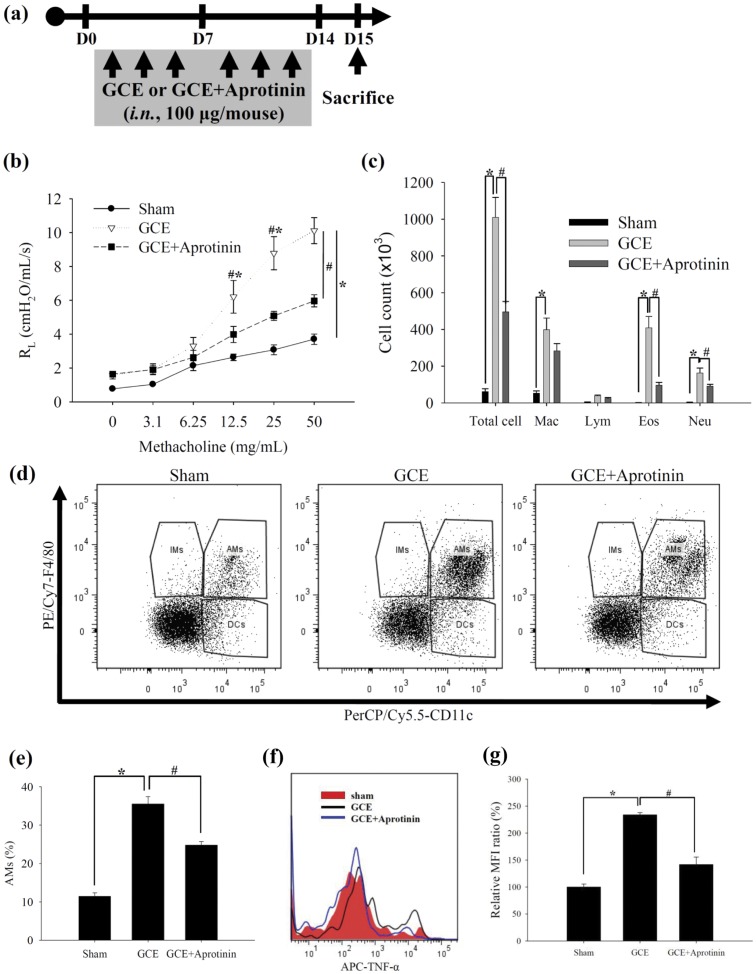
Serine protease in GCE develops pulmonary inflammation, alveolar macrophage infiltration and TNF-α expression. (a) Scheme for the inhibition of GCE protease activity of GCE-induced asthma model. (b) AHR and (c) BAL cell count. (d) Alveolar macrophage, interstitial macrophage, and dendritic cell population in the lung tissue. (e) Quantitative analysis of alveolar macrophage from “d” panel. (f) Intracellular expressions of TNF-α in the alveolar macrophages of the GCE-induced asthma experiment. (g) Relative MFI ratio in “f” panel. * indicates statistical significance between “Sham” and “GCE” (n = 5, p<0.05). ^#^ indicates statistical significance between “GCE” and “GCE+Aprotinin” (n = 5, *p*<0.05). All data are representative of three independent experiments. *AMs*, alveolar macrophages; *IMs*, interstitial macrophages; *DCs*, dendritic cells.

### Protease activity measurement

Protease activity of GCE was measured using a Protease Fluorescent Detection Kit (Sigma-Aldrich, St. Louis, MO) according to the supplier's recommendations [21]. A fluorescein isothiocyanate (FITC)-casein and GCE were prepared and the fluorescence intensity was recorded by Luminescence Spectrometer (Perkin-Elmer, Oak Brook, IL).

**Figure 6:**
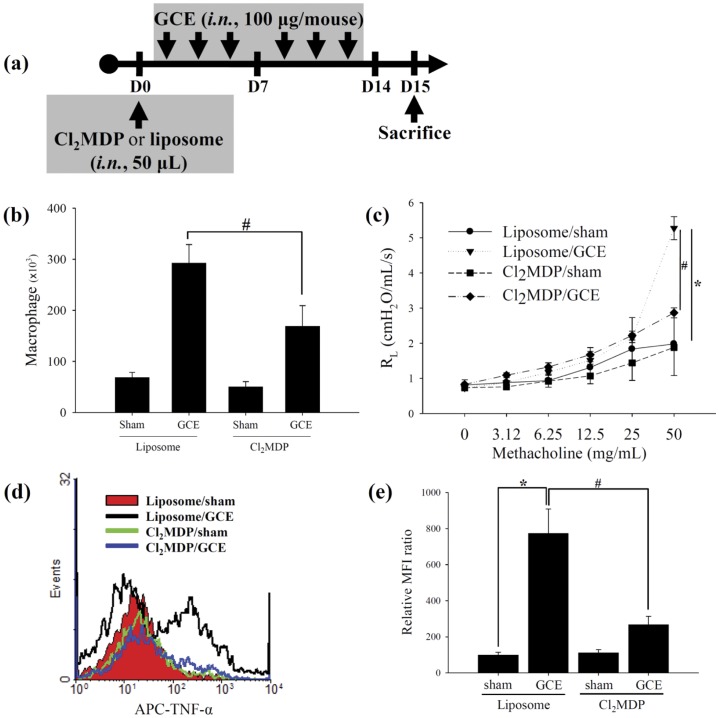
Alveolar macrophages are the major source of TNF-α in the GCE-induced asthma model. (a) Scheme for the alveolar macrophage depletion of GCE-induced asthma model. *Cl_2_MDP*, Cl_2_MDP-containing liposomes; *Liposome*, control liposomes. (b) Macrophages from BAL fluid and (c) AHR in alveolar macrophage-depleted animals. (d) Intracellular TNF-α production in lung tissue. (e) Relative MFI ratio in “d” panel. * indicates statistical significance between “Liposome/sham” and “Liposome/GCE” (n = 5, *p*<0.05). ^#^ indicates statistical significance between “Liposome/GCE” and “Cl_2_MDP/GCE” (n = 5, *p*<0.05). All data are representative of three independent experiments. *Liposome/sham*, control liposome-treated group; *Liposome/GCE*, control liposome-treated GCE-treated group; *Cl_2_MDP/sham*, Cl_2_MDP-containing liposome-treated sham group; *Cl_2_MDP/GCE*, Cl_2_MDP-containing liposome-treated GCE-treated group.

### Cell culture

MH-S and RAW264.7 cells (ATCC, Manassas, VA) were stimulated with GCE, ENMD-1068 (Enzo Life Sciences, Farmingdale, NY), aprotinin, or polymixin B (PMB; Sigma-Aldrich). The cells were cultured in Dulbecco's modified Eagle medium supplemented with 10% fetal bovine serum and 100 U/mL penicillin-streptomycin.

### Liposome-encapsulated Cl_2_MDP

Liposomes encapsulated with dichloromethylenediphosphonic acid disodium salt (Cl_2_MDP; Sigma-Aldrich) were prepared as previously described [22]. Briefly, 86 mg phosphatidylcholine and 8 mg cholesterol were dissolved in 10 mL chloroform or 4 mL PBS, and a lipid film was manufactured by low vacuum rotary evaporation. The suspension, which was dissolved in either 10 mL Cl_2_MDP or 4 mL PBS, was kept at room temperature for 2 hours and then sonicated for 5 minutes. The suspensions were then centrifuged at 100,000×*g* for 30 minutes to remove free Cl_2_MDP. The Cl_2_MDP-containing liposomes and the control liposomes were resuspended in 2 mL PBS.

### AHR measurement

AHR was measured as previously described [23]. Mice were anesthetized (pentobarbital sodium, intraperitoneally), ventilated (*flexiVent 5.1*®; SCIREQ, Montreal, Canada) and challenged with a saline aerosol followed by increasing concentrations of methacholine (MCh; Sigma-Aldrich). Aerosols were generated with an ultrasonic nebulizer (Omron Healthcare, Kyoto, Japan) and delivered to the inspiratory line of the *flexiVent* using a bias flow of medical air.

### BAL fluid

Bronchoalveolar lavage (BAL) fluid was obtained as previously described [23]. To collect BAL fluid, the lungs were lavaged with 1 mL Hank's balanced salt solution (HBSS) via the tracheostomy tube. Total cell numbers were counted with a hemocytometer. After the procedure, BAL fluid was centrifuged at 1,500×*g* for 3 minutes at 4°C, and then smears of BAL cells were prepared by cytocentrifugation (Cytospin3, Thermo, Billerica, MA) at 1,000 rpm for 3 minutes. BAL cells were stained with Hemacolor Staining Kit (Merck, Darmstadt, Germany) counted, and classified as neutrophils, eosinophils, lymphocytes, or macrophages.

### Lung homogenate

For assessment of cytokine levels, lung tissues were homogenized in 20 mL/g tissue protein extraction reagent (Thermo Fisher Scientific Inc., Rockford, IL) using a tissue homogenizer (Biospec Products, Bartlesville, OK). Homogenates were incubated at 4°C for 30 min and then centrifuged at 1,000×*g* for 10 min. Supernatants were collected, passed through a 0.45-micron filter (Gelman Sciences, Ann Arbor, MI), and then stored at −70°C for assessment of cytokine levels.

### Immunocytohistochemistry

Immunofluorescence staining of PAR-2 and TNF-α in Raw 264.7 cells and lung tissues was examined by confocal laser scanning microscopy (LSM700, Carl Zeiss, Jena, Germany). Cytospin-fixed RAW 264.7 cells or formalin-fixed, paraffin-embedded lung tissues were stained with FITC anti-mouse PAR-2 (SAM11, Santa Cruz Biotechnology, Santa Cruz, CA), phycoerythrin (PE) anti-mouse CD11b, and allophycocyanin (APC) anti-mouse TNF-α (BD Pharmingen^TM^, San Jose, CA) at 4°C for 30 minutes. After staining, the samples were washed and observed under confocal laser scanning microscopy with excitation wavelengths of 493, 565, and 645 nm and emission wavelengths of 525, 575, and 660 nm, respectively.

### Intracellular cytokine staining

Intracellular cytokine staining was performed using a Cytofix/Cytoperm kit (BD Biosciences, San Diego, CA) according to the supplier's recommendations. The cells were stained with PE/Cy7-anti-mouse CD4, PE-anti-mouse CD11b, PerCP/Cy5.5-anti-mouse CD11c and PE/Cy7-anti-mouse F4/80, permeabilized, and stained intracellularly with FITC-anti-mouse IFN-γ, PE-anti-mouse IL-5, APC-anti mouse IL-17, APC-anti-mouse TNF-α (BD Pharmingen), FITC- and PE-anti-mouse PAR-2 (Santa-Cruz Biotechnology).

### Flow cytometric analysis

We performed multicolor-flow cytometric analysis (LSRII; BD Biosciences). The data were analyzed using FACSDiva (BD Biosciences) or FlowJo ver.7.6.2 (Three Star, Ashland, OR) and expressed as a percentage value or mean fluorescence intensity (MFI). The relative-MFI ratio was calculated relative to the control group.

### ELISA

TNF-α, IFN-γ, IL-5 and IL-13 were detected by enzyme-linked immunosorbent assay (ELISA) with a DuoSet® ELISA (R&D Systems, Minneapolis, MN). Immunoglobulin (Ig)E was detected with an IgE ELISA set (BD Biosciences).

### Pulmonary pathology analysis

Periodic Acid-Schiff (PAS) and Masson's Trichrome staining were performed in the formalin-fixed/paraffin-embedded lung tissues. Tissue sections were examined with an Olympus BX40 microscope in conjunction with an Olympus U-TV0.63XC digital camera (Olympus Corp., Melvile, NY). Images were acquired using DP Controller and Manager software (Olympus Corp.). PAS^+^cells per millimeter of bronchial basement membrane (mmBM) and Trichrome^+^pixels per total area (%) were measured by MetaMorph 4.6 (Universal Imaging, Downingtown, PA).

### Statistical analysis

The data are expressed as mean±standard error. Statistical analyses were measured using SPSS ver.12.0 (Chicago, IL). Paired groups were compared using the Student's *t*-test, and all differences were considered significant at *p*<0.05.

## Results

### Serine protease activity of GCE activates PAR-2 in macrophages

German cockroaches exhibit protease activity that includes serine- and cysteine-degrading enzymes. We measured the protease activity in GCE on a FITC-casein substrate. Compared to the typical standard curve of trypsin from 0.16 µg/mL to 20 µg/mL on the FITC-casein substrate, one milligram of the GCE contained protease activity that was equivalent to 21.87 μg/mL trypsin (Figure 1A and 1B). To identify the specific protease activity in GCE, GCE was incubated with several protease inhibitors that inhibited serine or cysteine protease classes. When GCE was incubated with aprotinin-a serine protease inhibitor, E-64-a cysteine protease inhibitor, phenylmethanesulfonyl fluoride (PMSF)-a serine/cysteine protease inhibitor, and soybean trypsin inhibitor (STI)-a trypsin inhibitor, each protease activities were decreased. In addition, one of these inhibitors, aprotinin, markedly reduced protease activity in the GCE (Figure 1B).

To determine PAR-2 immunolocalization following GCE stimulation in the mouse macrophages, alveolar macrophage cell-lines (MH-S cells) and peritoneal macrophage cell-lines (RAW264.7 cells) were incubated with either GCE or a combination of GCE and aprotinin. GCE stimulation led to internalization of the PAR-2 in the MH-S cells, while the cells incubated with a combination of GCE and aprotinin revealed similar patterns of control, as visualized by confocal imaging of intracellular staining (Figure 1C) and cell surface staining samples (Figure S1). In the RAW264.7 cells, PAR-2 expression of the cell surface decreased following GCE stimulation, but the cells incubated with a combination of GCE and aprotinin revealed similar to the control (Figure S2)

To confirm a specific interaction between the GCE and the PAR-2, MH-S cells were incubated with an ENMD-1068, a novel selective PAR-2 antagonist [24], [25], [26], and then stimulated with GCE. The intracellular expression of PAR-2 and TNF-α was markedly inhibited by ENMD-1068 (Figure 1D–G).

### GCE induces TNF-α production in macrophages

To examine whether PAR-2 activation by serine proteases within GCE induces inflammation via macrophages, we studied TNF-α production and secretion in the MH-S and RAW264.7 cells. Intracellular TNF-α levels were significantly increased in GCE- stimulated MH-S cells, but these levels were not increased when GCE protease activity was inhibited by aprotinin. In GCE+PMB-stimulated condition, the endotoxin level less than 0.1 EU/mL in GCE had no effect in GCE-stimulated cells (Figure 2A and 2B). These results showed that serine protease but not endotoxin in GCE is critical for TNF-α production. The culture supernatants from cells grown under each condition revealed similar patterns of TNF-α production (Figure 2C). The results of RAW264.7 cells revealed similar to the MH-S cells (Figure S3).

To identify the kinetics of PAR-2 and TNF-α expression during GCE stimulation process, GCE was administered intranasally to BALB/c mice 3 times per week for 2 weeks (short-term GCE exposure model; Figure 2D). Intracellular PAR-2 and TNF-α levels of alveolar macrophages (CD11c^+^ and F4/80^+^ cells) were increased continuously for up to 2 weeks (Figure 2E–2G).

According to the immunohistochemistric analysis, TNF-α accumulation of macrophages were increased in the lung tissues of long-term GCE exposure model (3 times per week for 4 weeks; Figure S4).

### GCE promotes pulmonary inflammation through the release of TNF-α

To determine whether GCE promotes allergic asthma-like symptoms, we used a long-term GCE exposure model (Figure 3A). In an AHR assay, mice that received GCE developed pulmonary resistance based on the requirement for increasing doses of MCh inhalation (Figure 3B). The mice receiving GCE exhibited inflammatory cell infiltration in the BAL fluid, and the majority of the cells in the BAL fluid were macrophages, lymphocytes, and neutrophils (Figure 3C). Intracellular TNF-α production and secretion of the macrophages were increased in the long-term GCE exposure model (Figure 3D–3F).

To confirm the helper T cell differentiation during GCE-stimulation, intracellular and secreted levels of different cytokines in CD4^+^ T cells were measured. The intracellular levels of IFN-γ, IL-5 and IL-17 were increased in the CD4^+^ T cells of the long-term GCE exposure mice (Figure S5A and S5B).IFN-γ, IL-5, IL-10, IL-13 and IL-17 production in lung, and serum IgE levels were also increased in this model (Figure 4A–4D; Figure S5C). Histologically, goblet cell hyperplasia and collagen deposition in the peri-bronchiolar area were exacerbated in the long-term GCE exposure model as compared to controls (Figure 4E–4H).

To confirm the role of TNF-α in the long-term GCE exposure model, we blocked TNF-α in these animals. We intravenously injected goat anti-mouse TNF-α polyclonal antibody (10 µg/mouse, R&D Systems) or goat IgG (10 µg/mouse, R&D Systems) into the animals intravenously 12 hours before the first, 7^th^, 14^th^, and 21^st^ administrations of GCE (Figure 3A). The development of AHR and airway inflammation following GCE administration was attenuated by the systemic neutralization of TNF-α in these mice (Figure 3B and 3C). TNF-α blockade reduced intracellular TNF-α production in the macrophages as well as TNF-α secretion from the lung tissues of the long-term GCE exposure mice (Figure 3D–3F). TNF-α blockade also decreased Th2 cytokine production, serum IgE levels, goblet cell hyperplasia, and peri-bronchial fibrosis in the long-term GCE exposure model (Figure 4).

To confirm the effect of TNF-α blockade, as a therapeutic agent, we blocked TNF-α just before the last challenge in a short-term GCE exposure model. In this model, both AHR and inflammatory cell infiltration in the BAL fluid also improved (Figure S6), and kinetically increased of PAR-2 and TNF-α levels of alveolar macrophages were diminished in the TNF-α blockade group (Figure 2E–2G).

### Serine protease activity of GCE develops allergic pulmonary inflammation

We identified whether the serine protease activity of GCE has a critical role in the pulmonary allergic inflammation in the short-term GCE exposure model. We incubated GCE with aprotinin (100 µg) and administered the GCE+aprotinin intranasally to the mice 3 times per week for 2 weeks (Figure 5A). When the serine protease activity of GCE was inhibited by aprotinin, AHR (Figure 5B) and airway inflammation (Figure 5C) was attenuated. The inhibition of GCE protease activity reduced the infiltrations of alveolar macrophages (CD11c^+^ and F4/80^+^ cells; Figure 5D and 5E) and the intracellular TNF-α production of the alveolar macrophages (TNF-α^+^, CD11c^+^ and F4/80^+^ cells; Figure 5F and 5G), whereas it did not affect the infiltration of interstitial macrophages (CD11c^−^ and F4/80^+^ cells) nor dendritic cells (CD11c^+^ and F4/80^−^ cells) in the short-term GCE exposure model (Figure 5D and Figure S7).

### Activated alveolar macrophages and their TNF-α production promote GCE-induced pulmonary inflammation

According to our *in vitro* and *in vivo* studies, we demonstrated that serine protease activity in the GCE stimulated macrophages, which are the major source of TNF-α production. To examine the function of alveolar macrophages in the short-term GCE exposure models, we intranasally treated mice with Cl_2_MDP-containing liposomes (50 µL/mouse) or control liposomes (50 µL/mouse) intranasally 12 hours before the first administration of GCE and then selectively eliminated alveolar macrophages (Figure 6A). The number of macrophages in the BAL fluid and the AHR induced by the GCE treatment were significantly reduced in the alveolar macrophage-depleted mice compared to the non-depleted animals (Figure 6B and 6C). Intracellular TNF-α levels in the lung cells of short-term GCE exposure mice were significantly decreased by depletion of the alveolar macrophages (Figure 6D and 6E).

## Discussion

In this study, we showed that GCE contains strong serine protease activity and that the serine proteases activated and internalized PAR-2 in GCE-stimulated MH-S and RAW264.7 cells. Upon binding of the serine protease to PAR-2 in a cell, an N-terminal bond at Arg^34^-Ser^35^ of PAR-2 is cleaved, and the newly exposed N-terminal sequence activates the receptor as a “tethered ligand”. This PAR-2 activation results in G protein-mediated intracellular signaling and triggers release of inflammatory cytokines in the airway [27]. The receptor is then internalized for recycling or lysosomal degradation [28]. PAR-2 can also be activated by tryptase from mast cells, and thus, the presence of protease activity may not be essential for the other inhalant allergens. In fact, cat dander and dog dander allergens are well-known inhalant allergens for asthma or allergic rhinitis, but these allergens do not contain protease activities [29].

Asthma is the common chronic inflammatory disease of the airway and one of the causes of asthma, indoor allergen is associated with asthma development [30]. Mouse models of asthma induced by allergen exposure have been developed and characterized by the reproducible AHR, inflammation and remodeling. Both short- and long-term exposure of allergens have caused the development of allergic asthma [31]. In our study, both short- and long-term exposure to GCE without additional adjuvants induced allergic asthma-like lesions in BALB/c mice, but the types of pulmonary inflammation between these groups were different. In long-term GCE exposure mice, neutrophils rather than eosinophils were more infiltrated in the BAL fluid, and IL-17-produced helper T cells were increased in the lung tissue. Previously, other groups have shown that longer exposure to allergens can lead to tolerance [32], [33], [34] and Th17-mediated airway remodeling [35]. Our results partially suggest that a long-term exposure to GCE has the possibility to induce prominent IL-10 and IL-17 production. Indeed, GCE exposure caused AHR, Th2 cytokine production, and serum IgE secretion. Histologically, goblet cell hyperplasia and fibrosis in the peri-bronchiolar area were exacerbated in the GCE-induced asthma model. These results show that the active serine protease of the GCE may boost allergen-mediated immune responses without the aid of adjuvant, suggesting that the protease activities in cockroaches may actually have adjuvant effects to sensitize the animals to cockroach allergens [36]. These features are also found in house dust mite allergen-induced asthma models, as these allergens also possess strong serine and cysteine protease activities [37], and other substances acting as adjuvants [38]. Recently, some investigators have reported that cockroach has cysteine protease [39], [40] and it can also activate PAR-2 pathway [41], [42]. Protease inhibition assay in our results also revealed that GCE sufficiently contained serine protease as well as cysteine protease. However, it has also been reported that cysteine protease in cockroach does not have a sufficient role in the induction of allergic airway inflammation [43] and pro-inflammatory effect on airway epithelial cells to cockroach [44].

Macrophages are a major cell type in lung tissue, and most of these cells exist in the pulmonary alveolus [45]. It involve in both the innate and adaptive immune responses and may be one of the major sources of IL-13 in asthma in mouse models [46]. Furthermore, activation of alveolar macrophages by Th2 cytokines results in stimulation of the allergic immune responses, airway inflammation [47], and pulmonary fibrosis [48].

Recently, Day and coworkers (2010) had investigated the relationship between serine protease, PAR-2, alveolar macrophages and TNF-α in German cockroach frass inhaled mice [49]. They also presented that the proteases in frass could induce the innate immune response in mice via activation of PAR-2 [43]. In this study, we hypothesized that PAR-2 activation of the alveolar macrophages and production of pro-inflammatory components, such as TNF-α, play a critical role in the GCE-induced asthma model. In addition to macrophages, PAR-2 is also expressed by epithelium, myocytes, neutrophils and T lymphocytes, and these type of cells may play important roles in sensitization, initiation and persistency of allergic inflammation [3], [4], [7], [8], [50]. In this study, we showed that GCE induced considerable amount of TNF-α production in alveolar macrophages via PAR-2 pathway *in vitro* cross-sectional and *in vivo* kinetic study; however, other investigators showed that PAR-2 may play a protective role against bronchoconstriction or AHR in rat, mouse, and guinea pig by the release of a cyclooxygenase product from the epithelium [15]. So it is still controversial that PAR-2 expression mediates either proinflammatory or anti-inflammatory activities in inflammatory lung disorders [51].

TNF is a pleiotrophic cytokine for the innate immune response and implicated in the mechanisms of several inflammatory diseases, such as chronic asthma, inflammatory bowel disease, and rheumatoid arthritis, and it is produced mainly by activated macrophages in various tissues [18]. This cytokine is a chemoattractant for eosinophils and neutrophils, increases expression of adhesion molecules in epithelium and endothelium, activates T lymphocytes, and enhances the contractility and proliferation of airway smooth muscle cells [18]. PAR-2 induced TNF-α may activate dendritic cells and enhance allergen uptake by the cells and ultimately promote allergen sensitization instead of tolerance [4]. This notion is supported by the findings that PAR-2 knock-out mouse has developmental defects in dendritic cells [52]. In many studies, anti-TNF-α therapy has been regarded as an attractive strategy for the management of asthma [18], [20]. In support of this idea, we found that neutralization of TNF-α attenuated production of Th1 and Th2 cytokines, specific IgE, TNF-α and PAR-2 expression by alveolar macrophages, and airway remodeling, and these features can be seen even it was administered after establishment of GCE induced allergic inflammation, suggesting that anti-TNF-α treatment may be an important candidate for treatment of asthma.

The fact that the alveolar macrophage mediated TNF-α production is crucial for the development of allergic asthma like features was supported by results from alveolar macrophage-depletion using Cl_2_MDP-containing liposomes. Intranasal treatment with Cl_2_MDP-containing liposomes [53], [54] can selectively deplete alveolar macrophages, whereas macrophages in the interstitial zone and other monocytes were not affected [16], [22]. Following the alveolar macrophage depletion, physiological aggravation of the lung tissue in the GCE-asthma model mice was improved, and TNF-α-producing cells were reduced in the area. Our results are consistent with the previous reports showing that alveolar macrophages are a major source of TNF-α in an allergic asthma model [4], [18], [49]. TNF-α also can be produced by endotoxin, but in this study we used endotoxin depleted GCE which contain less than 0.01 EU/mL.

Our results suggest that the serine protease activity in GCE induces TNF-α production by macrophages via the PAR-2 pathway. Upon intranasal administration of GCE into mice, allergic asthma-like features developed as a result of alveolar macrophage activation and TNF-α production in the lung tissue. This study, however, does not provide a specific mechanism for PAR-2 activation in the GCE-induced asthma model, and the precise mechanisms by which serine protease of GCE mediates allergic airway inflammation via macrophages remain to be elucidated.

## Supporting Information

Figure S1 **PAR-2 internalization following activation with GCE and/or aprotinin in MH-S cells.** Cell-surface expression of the PAR-2 is visualized by confocal imaging of cell-surface staining. All data are representative of three independent experiments.
(DOC)

Figure S2 **PAR-2 internalization following activation with GCE and/or aprotinin in RAW264.7 cells.** Cell-surface expression of the PAR-2 is visualized by confocal imaging of cell-surface staining. All data are representative of three independent experiments.
(DOC)

Figure S3 **GCE induces TNF-α production in RAW264.7 cells.** (a) TNF-α production in RAW264.7 cells incubated with GCE, GCE+PMB, or GCE+aprotinin. (b) Relative MFI ratio in “a” panel. (c) TNF-α secretion in the culture supernatant of “a” panel. * indicates statistical significance between “Control” and “GCE” (n = 3, *p*<0.05). ^#^ indicates statistical significance between “GCE” and “GCE+Aprotinin” (n = 3, *p*<0.05). All data are representative of three independent experiments.
(DOC)

Figure S4 **GCE induces TNF-α production in lung tissue of mice.** (a) Scheme for a long-term GCE exposure model. (b) TNF-α expression in the macrophages of the lung tissue. All data are representative of three independent experiments.
(DOC)

Figure S5 **Intracellular cytokines expression in a long-term GCE exposure model.** (a) Intracellular expressions of IFN-γ, IL-5 and IL-17 from CD4^+^ T cells in the lung tissue of the long-term GCE exposure model. (b) Cytokine^+^ T cells in “a” panel. (c) IL-10 and IL-17 production in the lung homogenate of the long-term GCE exposure model. * indicates statistical significance between “Sham” and “GCE” (n = 5, *p*<0.05).
(DOC)

Figure S6 **Therapeutic effect of TNF-α blockade in a short-term GCE exposure model.** (a) AHR and (b) BAL cell count. * indicates statistical significance between “Sham” and “GCE” (n = 5, p<0.05). ^#^ indicates statistical significance between “GCE” and “a-TNF” (n = 5, *p*<0.05). *R_L_*, pulmonary resistance; *Mac*, macrophage; *Lym*, lymphocyte; *Eos*, eosinophil; *Neu*, neutrophil.
(DOC)

Figure S7 **Quantitative analysis of (a) interstitial macrophage and (b) dendritic cell population from GCE-induced asthma model.** All data are representative of three independent experiments. (n = 5, *p*<0.05) *IMs*, interstitial macrophages; *DCs*, dendritic cells.
(DOC)
