# Supplementary figures and images for: Alveolar Macrophages Play a Key Role in Cockroach-Induced Allergic Inflammation via TNF-α Pathway
Source: PLoS One. 2012 Oct 19;7(10):e47971. doi: 10.1371/journal.pone.0047971 (PMC3477122; doi:10.1371/journal.pone.0047971)

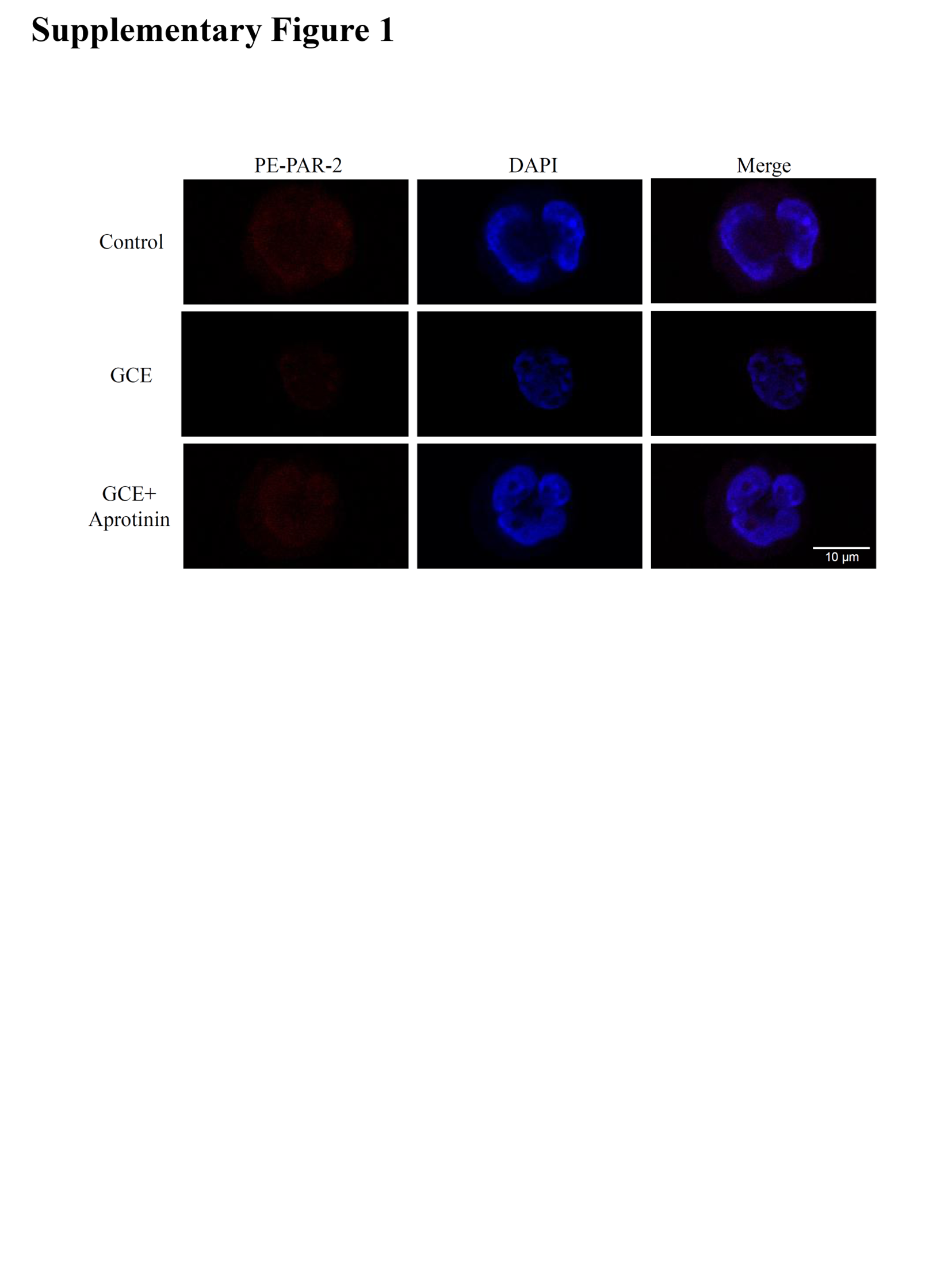

Supplement: Figure S1 — PAR-2 internalization following activation with GCE and/or aprotinin in MH-S cells. Cell-surface expression of the PAR-2 is visualized by confocal imaging of cell-surface staining. All data are representative of three independent experiments. (DOC) [file pone.0047971.s001.doc]

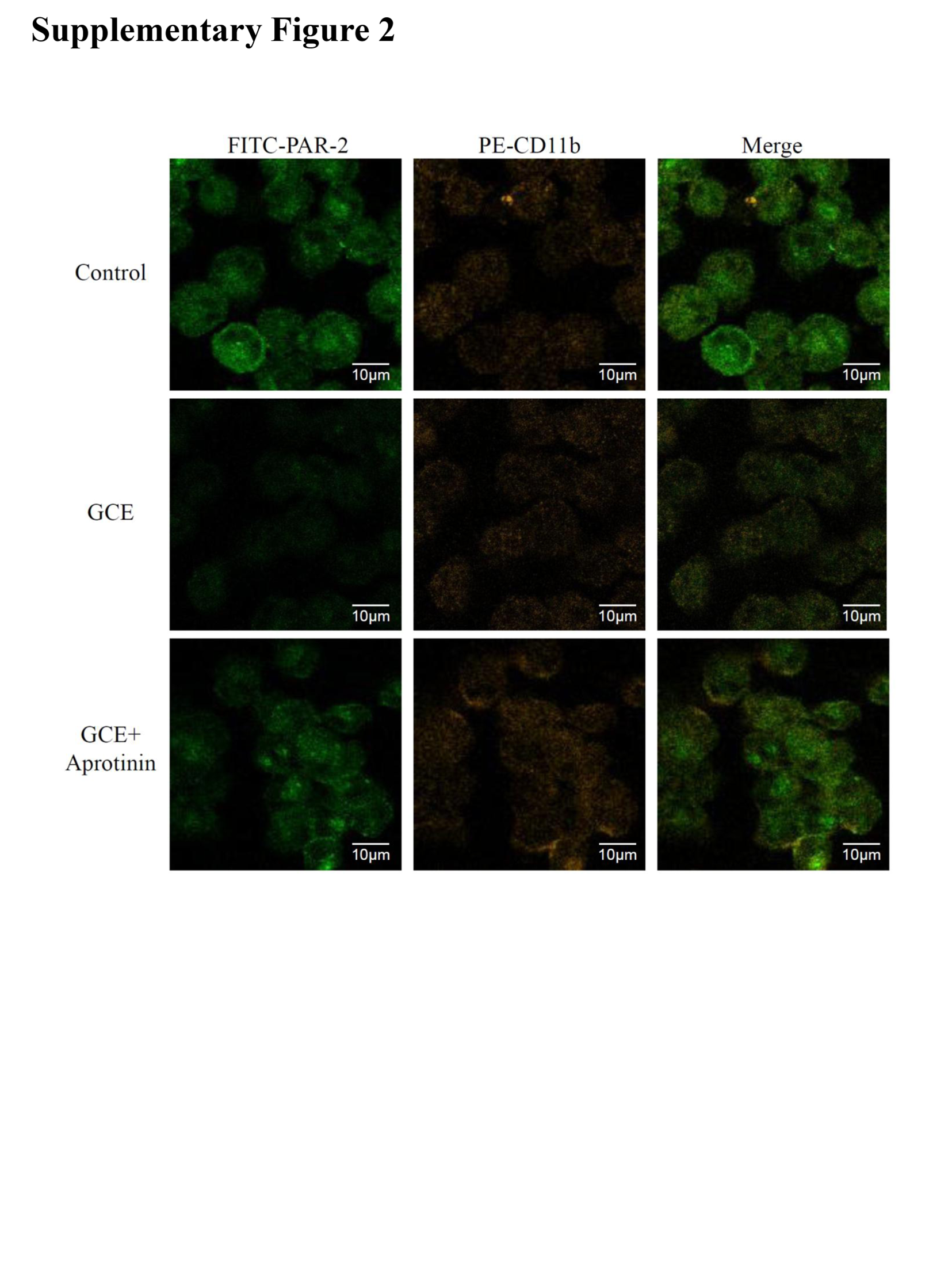

Supplement: Figure S2 — PAR-2 internalization following activation with GCE and/or aprotinin in RAW264.7 cells. Cell-surface expression of the PAR-2 is visualized by confocal imaging of cell-surface staining. All data are representative of three independent experiments. (DOC) [file pone.0047971.s002.doc]

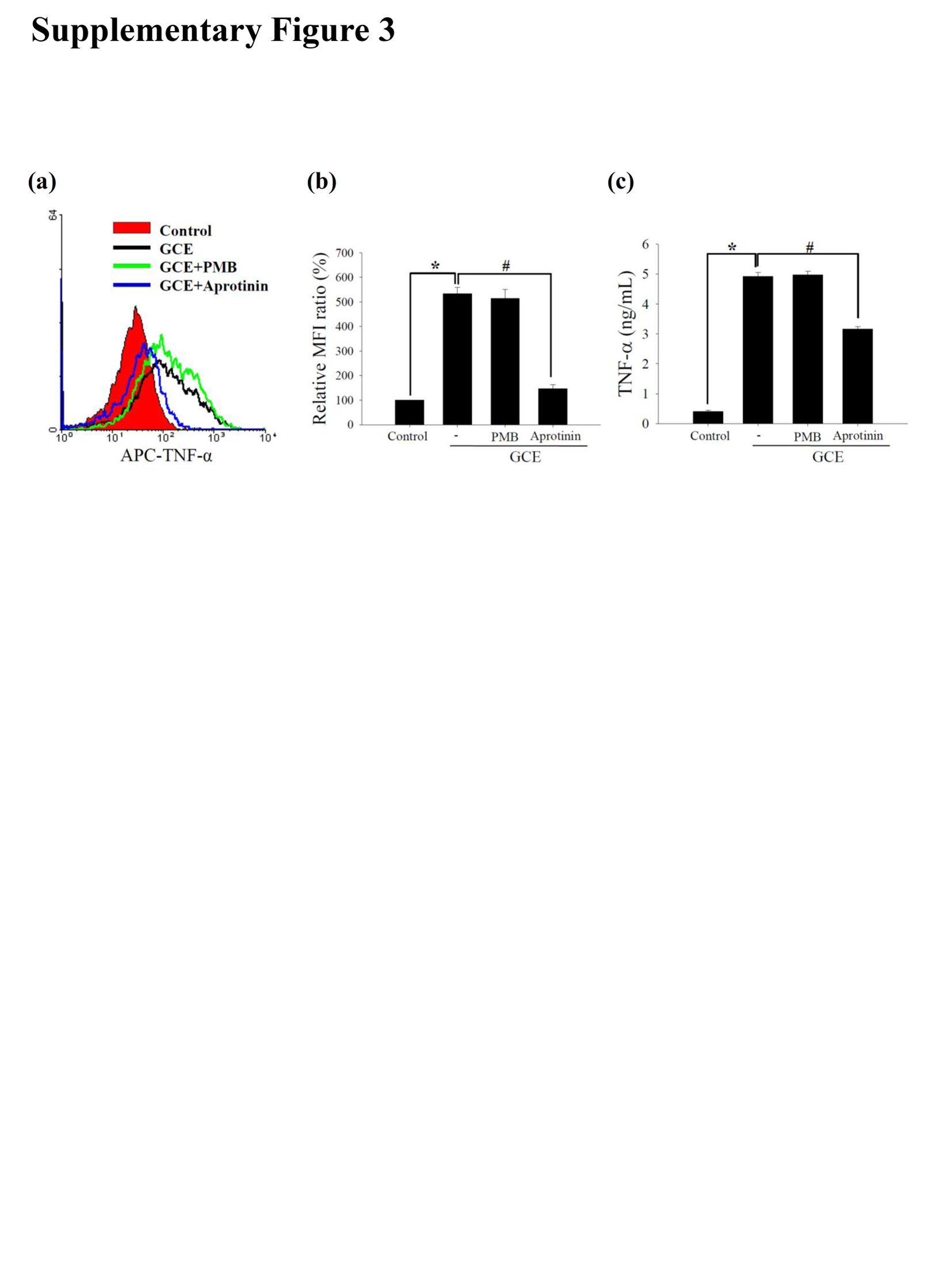

Supplement: Figure S3 — GCE induces TNF-α production in RAW264.7 cells. (a) TNF-α production in RAW264.7 cells incubated with GCE, GCE+PMB, or GCE+aprotinin. (b) Relative MFI ratio in “a” panel. (c) TNF-α secretion in the culture supernatant of “a” panel. * indicates statistical significance between “Control” and “GCE” (n = 3, p<0.05). # indicates statistical significance between “GCE” and “GCE+Aprotinin” (n = 3, p<0.05). All data are representative of three independent experiments. (DOC) [file pone.0047971.s003.doc]

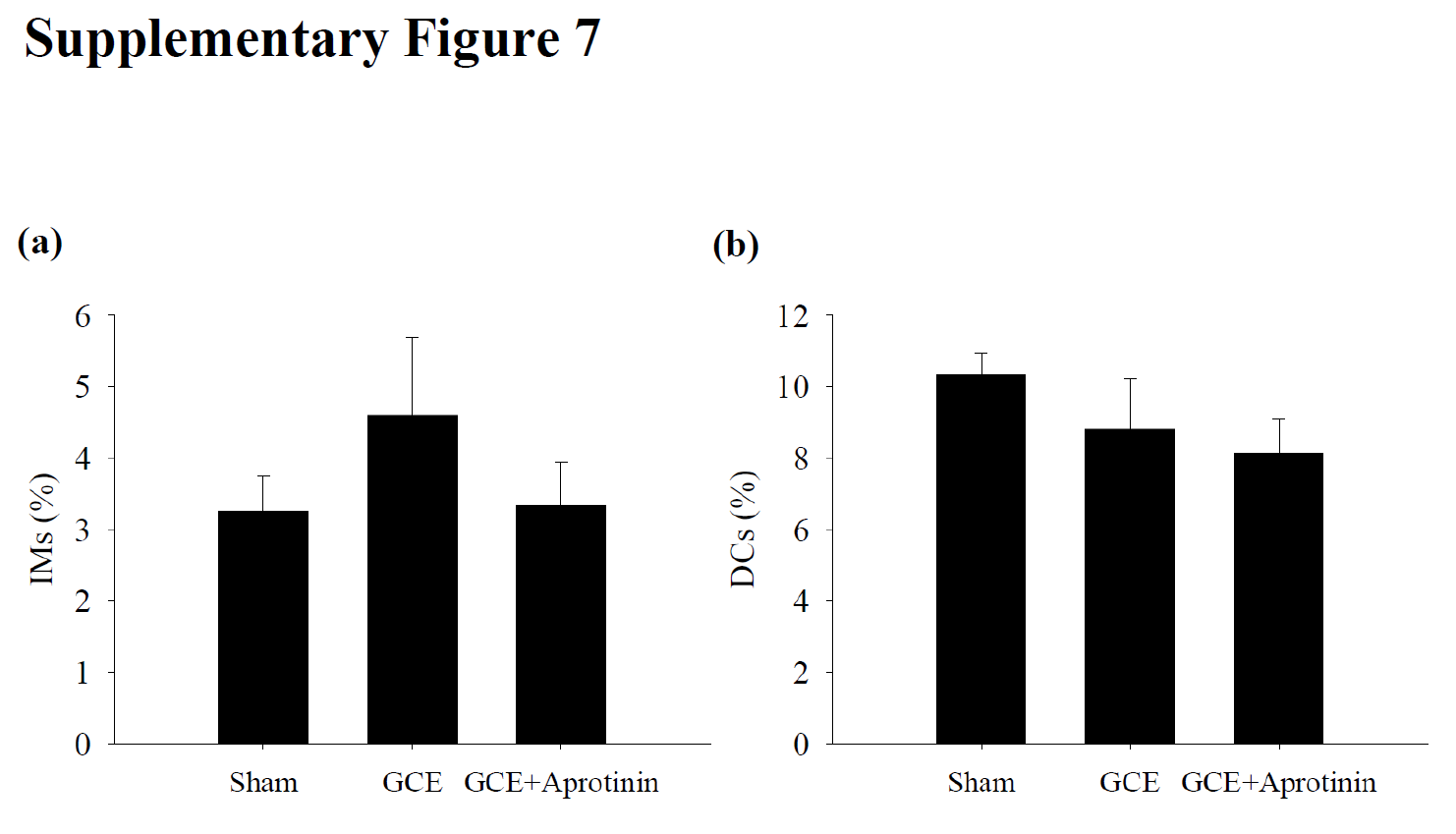

Supplement: Figure S7 — Quantitative analysis of (a) interstitial macrophage and (b) dendritic cell population from GCE-induced asthma model. All data are representative of three independent experiments. (n = 5, p<0.05) IMs, interstitial macrophages; DCs, dendritic cells. (DOC) [file pone.0047971.s007.doc]
