# Supplementary material for: Alveolar Macrophages Play a Key Role in Cockroach-Induced Allergic Inflammation via TNF-α Pathway
Source: PLoS One. 2012 Oct 19;7(10):e47971. doi: 10.1371/journal.pone.0047971 (PMC3477122; doi:10.1371/journal.pone.0047971)

Supplementary Figure 4

(a)

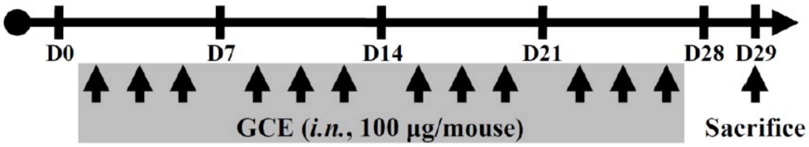

(b)

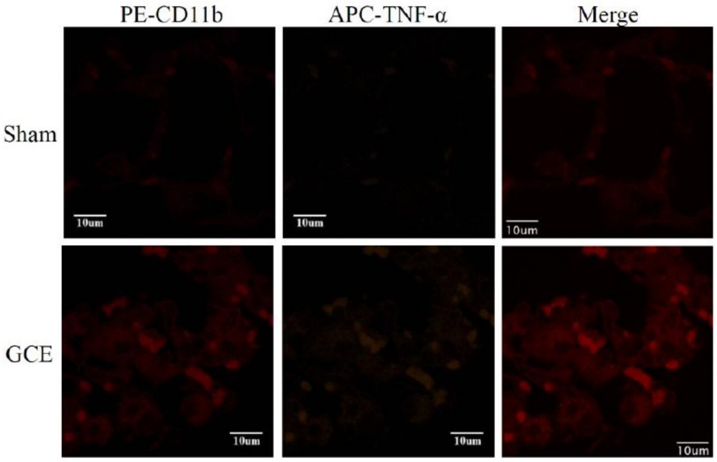

Supplement: Supplementary file 2 [file pone.0047971.s004.doc]
