# Supplementary material for: Alveolar Macrophages Play a Key Role in Cockroach-Induced Allergic Inflammation via TNF-α Pathway
Source: PLoS One. 2012 Oct 19;7(10):e47971. doi: 10.1371/journal.pone.0047971 (PMC3477122; doi:10.1371/journal.pone.0047971)

Supplementary Figure 5

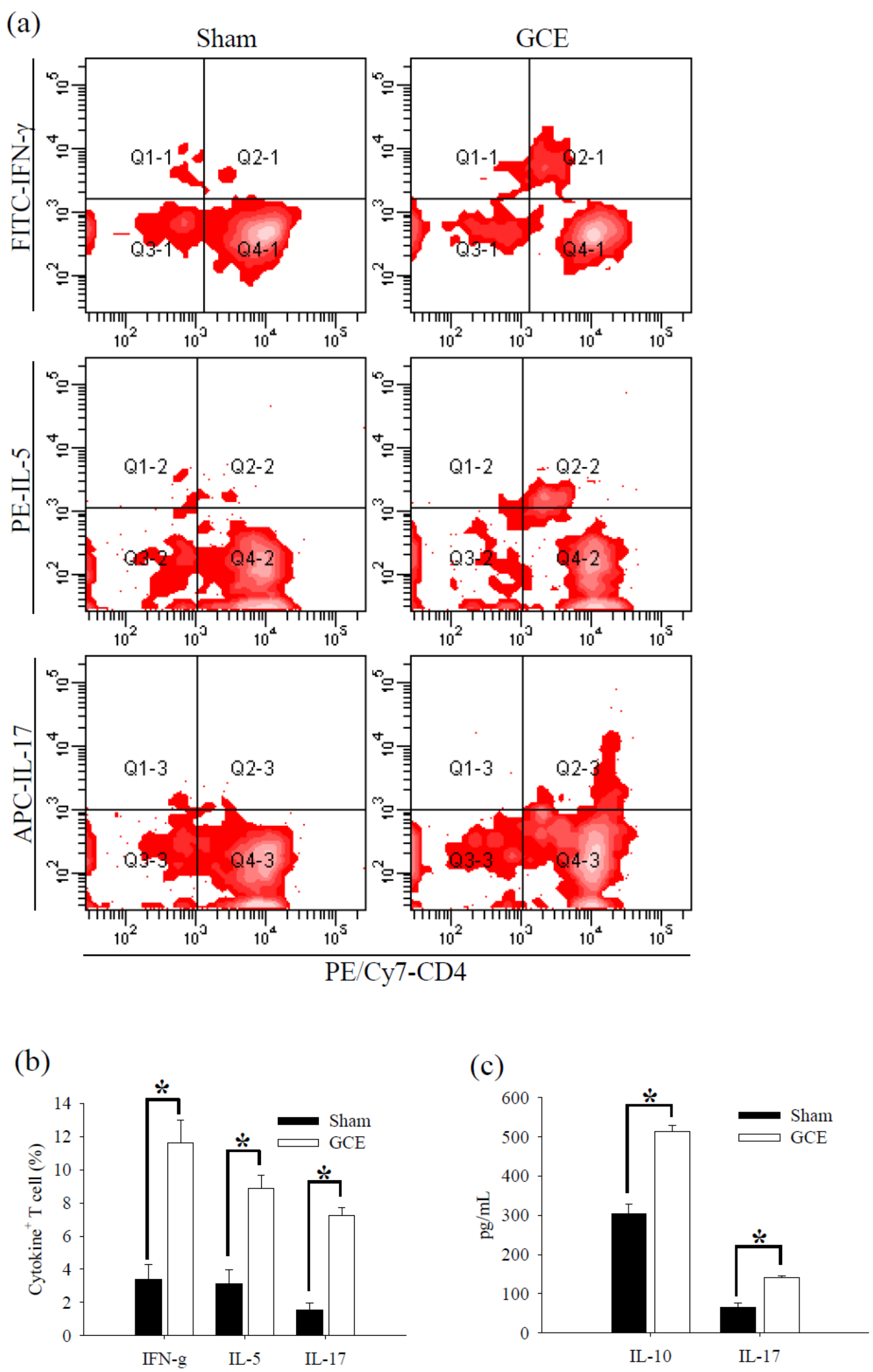

Supplement: Supplementary file 3 [file pone.0047971.s005.doc]
